# Pediatric Urinary Antimicrobial Resistance Patterns in a Tertiary Hospital of Northern Greece: A Retrospective Laboratory-Based Surveillance Study

**DOI:** 10.3390/antibiotics15080718

**Published:** 2026-07-24

**Authors:** Zafeiris Tsinaris, Theodouli Stergiopoulou, Paraskevi Mantzana, Areti Tychala, Olga Vasilaki, Georgia Kagkalou, Maria Stamou, Georgios Meletis, Efthymia Protonotariou, Assimina Galli-Tsinopoulou, Lemonia Skoura

**Affiliations:** 1 Department of Microbiology, AHEPA University General Hospital, School of Medicine, Aristotle University of Thessaloniki, 546 36 Thessaloniki, Greece; vimantzana@gmail.com (P.M.); protonotariou@auth.gr (E.P.);; 2 2nd Pediatric Department, AHEPA University General Hospital, Aristotle University of Thessaloniki, 546 36 Thessaloniki, Greece; stergiopouloulitsa@auth.gr (T.S.);

**Keywords:** pediatric urinary tract infection, antimicrobial resistance, antibiogram, *Escherichia coli*, *Klebsiella pneumoniae*, *Pseudomonas aeruginosa*, ESBL phenotype, carbapenem resistance

## Abstract

Background/Objectives: Antimicrobial resistance in pediatric urinary tract infections (UTIs) is a growing concern. We evaluated resistance patterns of *Escherichia coli*, *Klebsiella pneumoniae* and *Pseudomonas aeruginosa* urinary isolates in a tertiary care Greek hospital from 2015 through 12 September 2025. Methods: We retrospectively analyzed pediatric urinary isolates collected from 2015 through September 2025. Antibiotic analysis was restricted to EUCAST UTI-relevant organism-drug pairs. Resistance was summarized for the prespecified antibiotics, and presumptive ESBL and carbapenem resistance phenotypes were assessed. Results: Among 776 isolates (567 *E. coli*, 104 *K. pneumoniae*, 105 *P. aeruginosa*), *E. coli* showed the lowest resistance among non-carbapenem options to nitrofurantoin (0.4%), amikacin (1.2%), and gentamicin (7.8%), and the highest to trimethoprim (98.8%), ampicillin (53.5%), and trimethoprim-sulfamethoxazole (31.5%). *K. pneumoniae* had the lowest resistance among non-carbapenem options to amikacin (8.6%) and gentamicin (15.5%), with high cephalosporin resistance (oral cefuroxime 40.9%, intravenous cefuroxime 39.7%). Presumptive ESBL and carbapenem resistance in *K. pneumoniae* were 42.3% and 20.2%, respectively. Corresponding prevalences in *E. coli* were 12.4% and 0.7%. *P. aeruginosa* retained low resistance to meropenem (1.9%) and piperacillin-tazobactam (2.1%). No age or sex association with the presumptive resistance phenotypes remained significant after false-discovery-rate adjustment. Conclusions: *E. coli* showed very high ampicillin resistance, low resistance to gentamicin and amikacin, and moderate resistance to guideline-relevant cephalosporins. *P. aeruginosa* retained low resistance to key antipseudomonal agents. *K. pneumoniae* showed the highest burden of cephalosporin resistance and of presumptive ESBL and carbapenem resistance phenotypes. These data support guideline-based empirical coverage more strongly for *E. coli* than for *K. pneumoniae* in this setting.

## 1. Introduction

Urinary tract infections (UTIs) are among the most common bacterial infections in infancy and childhood [1,2,3,4,5,6]. They lead to substantial emergency department visits, frequent outpatient consultations and inpatient admissions, ultimately driving a large amount of pediatric antibiotic use [1]. However, physicians must often start empirical treatment before culture results are available [3,4,7].

This empirical choice is becoming increasingly difficult as antimicrobial resistance limits the available options. Large pediatric studies have shown increasing resistance to commonly used oral agents. Prior antibiotic exposure can increase the risk of a resistant infection for months, and prescribing practices have drifted toward broader-spectrum therapy in many settings [8,9,10,11]. Early effective therapy is crucial, as delayed antimicrobial therapy for febrile pediatric UTIs is associated with severe complications, including permanent renal scarring [12]. Therefore, up-to-date local resistance data are essential to guide empiric choices and not merely serve as descriptive statistics.

*Escherichia coli* remains the dominant pediatric uropathogen. However, other common Gram-negative organisms deserve explicit attention. Pediatric studies have shown that *Klebsiella pneumoniae* and *Pseudomonas aeruginosa* are particularly important, as they are more frequent in children with prior healthcare contact or structural urinary tract abnormalities. Patients needing urologic follow-up often harbor these organisms, and recurrent infections or recent antibiotic exposure can further increase their frequency. These organisms often carry broader resistance phenotypes than community-acquired *E. coli* [13,14].

Earlier Greek pediatric data also show regional and temporal variation in urinary resistance patterns [15,16]. This argues for local interpretation rather than reliance on pooled estimates alone. The same literature also points to the growing relevance of ESBL-associated pediatric urinary infection, where cephalosporin activity is reduced and oral options quickly disappear [17,18,19,20,21,22]. The study objectives were to (1) describe resistance to antibiotics relevant to pediatric UTI treatment; (2) estimate the prevalence of presumptive ESBL and carbapenem-resistance phenotypes; and (3) compare age and sex distributions across pathogens.

## 2. Results

### 2.1. Cohort Characteristics

The final analytic dataset contained 776 urinary isolates collected during 2015–2025, with 2025 treated as a partial year (samples up to 12 September 2025 were retrieved). Of these isolates, 567 were *E. coli*, 104 were *K. pneumoniae*, and 105 were *P. aeruginosa*. Over the study period, the relative distribution of these pathogens shifted significantly. The proportion of *E. coli* isolates increased from 53.1% in 2015 to 84.6% in 2025 (OR 1.12 per year, 95% CI 1.06–1.18; *p* = 0.0001, q = 0.0003). Conversely, the relative share of *K. pneumoniae* decreased from 21.9% to 7.7% (OR 0.92 per year, 95% CI 0.86–0.98; *p* = 0.015, q = 0.015), and the share of *P. aeruginosa* also decreased from 25.0% to 7.7% (OR 0.90 per year, 95% CI 0.83–0.97; *p* = 0.005, q = 0.008). (Supplementary Table S1).

*E. coli* accounted for most isolates. Isolates from female patients represented 65.8% (373/567) of *E. coli*, 50.0% (52/104) of *K. pneumoniae*, and 52.4% (55/105) of *P. aeruginosa*.

**Figure 1:**
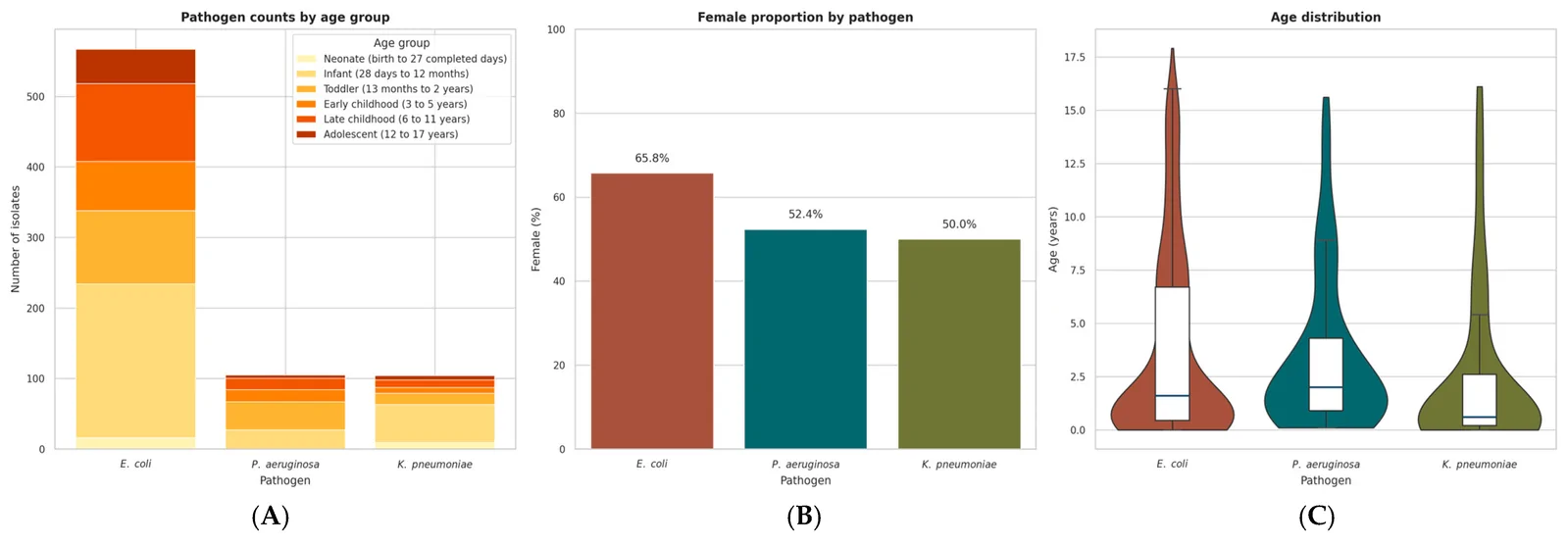
Cohort overview by pathogen. (**A**) Isolate counts stratified by age group. (**B**) Proportion of isolates from female patients by pathogen. (**C**) Violin plots showing age distributions by pathogen.

Patient age differed by pathogen (Kruskal–Wallis H = 25.43, *p* < 0.001, ε^2^ = 0.030). Children with *Klebsiella pneumoniae* isolates were younger (median 0.6 years, IQR 0.2–2.6) than those with *Escherichia coli* (1.6 years, IQR 0.4–6.7) or *Pseudomonas aeruginosa* isolates (2.0 years, IQR 0.9–4.3; both FDR-adjusted q < 0.001). Age did not differ significantly between the *E. coli* and *P. aeruginosa* groups (q = 0.288) (Figure 1).

Sex distribution also differed by pathogen (χ^2^ = 13.90, *p* < 0.001, Cramér’s V = 0.134), and the result was unchanged after all-time first-isolate filtering (χ^2^ = 13.84, *p* < 0.001, Cramér’s V = 0.136). *E. coli* had the highest proportion of isolates from female patients (65.8%), compared with *K. pneumoniae* (50.0%) and *P. aeruginosa* (52.4%) (Figure 1).

### 2.2. Resistance Patterns for Selected Antibiotics

Among non-carbapenem options for *E. coli*, resistance was lowest to nitrofurantoin (0.4%), amikacin (1.2%), and gentamicin (7.8%), whereas resistance was highest to trimethoprim (98.8%), ampicillin (53.5%), and trimethoprim-sulfamethoxazole (31.5%) (Table 1).

*Klebsiella pneumoniae* showed substantially higher resistance than *E. coli* across the adequately tested cephalosporins. Among non-carbapenem options, amikacin (8.6%) and gentamicin (15.5%) retained the greatest activity.

*Pseudomonas aeruginosa* retained low resistance across the restricted antipseudomonal set, including meropenem (1.9%) and piperacillin-tazobactam (2.1%).

### 2.3. Age-Stratified and Sex-Stratified Resistance Patterns

In addition to the overall descriptive resistance, we examined the raw resistance burden stratified by the patient’s age group and sex. To contextualize these descriptive percentages, we also provide nominal *p*-values derived from our multivariable logistic regression models. These models evaluate age (as a continuous variable) and sex (adjusted for age) as predictors of resistance for each specific antibiotic.

When observing resistance by Munich Age Classification System (MACS) categories (Neonate, Infant, Toddler, Early Childhood, Late Childhood, Adolescent), we found notable variation in the absolute burden of resistance. In *E. coli*, ampicillin resistance was higher among infants at 59.1% (127/215) and lowest among adolescents at 33.3% (16/48) (age effect *p* = 0.0406). In *K. pneumoniae*, resistance to key cephalosporins was highest among infants and late childhood patients; for example, cefotaxime resistance was 44.2% (23/52) in infants and 45.5% (5/11) in late childhood, though the smaller sample sizes in the older age categories limit precision (age effect *p* = 0.22).

Stratification by sex also revealed differing raw burdens of resistance. In *E. coli*, male patients generally exhibited a higher raw percentage of resistant isolates across several important antibiotics compared to female patients. For example, male ampicillin resistance was 58.9% (113/192) compared to 50.7% (185/365) in females (sex effect *p* = 0.44). Cefotaxime resistance in *E. coli* was 17.6% (33/187) in males and 10.0% (35/351) in females (sex effect *p* = 0.07). Similarly, trimethoprim-sulfamethoxazole resistance was 35.1% (68/194) in males versus 29.6% (110/371) in females (sex effect *p* = 0.06). In *K. pneumoniae* and *P. aeruginosa*, resistance rates between males and females were more variable and less consistently skewed, partly due to the smaller and more evenly balanced sample sizes. (Supplementary Tables S2–S4, Supplementary Figures S1–S3).

After FDR correction in the antibiotic-level multivariable models, two associations remained significant. Increasing age was associated with higher *E. coli* trimethoprim-sulfamethoxazole resistance (OR 1.079 per year, 95% CI 1.035–1.125; *p* = 0.0003, q = 0.0054), and male sex was associated with higher *E. coli* tobramycin resistance (OR 3.287, 95% CI 1.583–6.825; *p* = 0.0014, q = 0.0252) (Supplementary Table S15).

### 2.4. Phenotype-Level Resistance Burden

**Figure 2:**
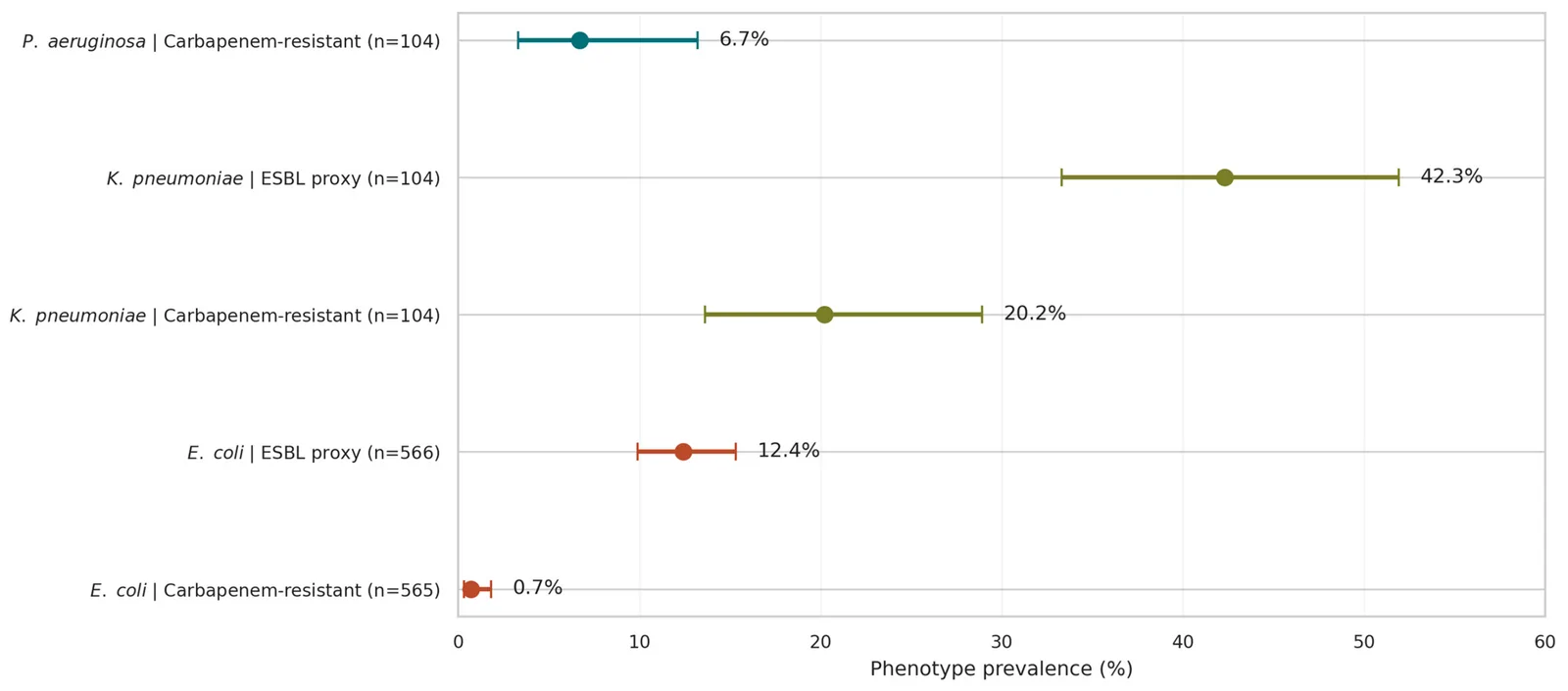
Presumptive ESBL and carbapenem-resistance phenotype prevalence by pathogen. Error bars show Wilson 95% confidence intervals. Phenotype denominators include isolates with at least one valid result among the phenotype-defining agents. Colors identify pathogens: *E. coli* (rust), *K. pneumoniae* (olive), and *P. aeruginosa* (teal).

*Klebsiella pneumoniae* showed higher presumptive ESBL and carbapenem resistance prevalence than *E. coli* (Figure 2; Supplementary Table S5).

Of the 567 *E. coli* isolates, one lacked a valid result among the cephalosporins defining the presumptive ESBL phenotype, and two lacked a valid result among the carbapenems defining the carbapenem resistance phenotype. The corresponding denominators were therefore 566 and 565. One of the 105 *P. aeruginosa* isolates lacked a valid result for both imipenem and meropenem, yielding a carbapenem resistance phenotype denominator of 104.

In the sensitivity analysis, the stricter ESBL proxy required resistance to at least two third- or fourth-generation cephalosporins among isolates with at least two valid results. Prevalence was 9.2% (52/565; 95% CI 7.1–11.9) in *E. coli* and 34.6% (36/104; 95% CI 26.2–44.2) in *K. pneumoniae* (Supplementary Table S14).

### 2.5. Age and Age-Adjusted Sex Analyses

**Figure 3:**
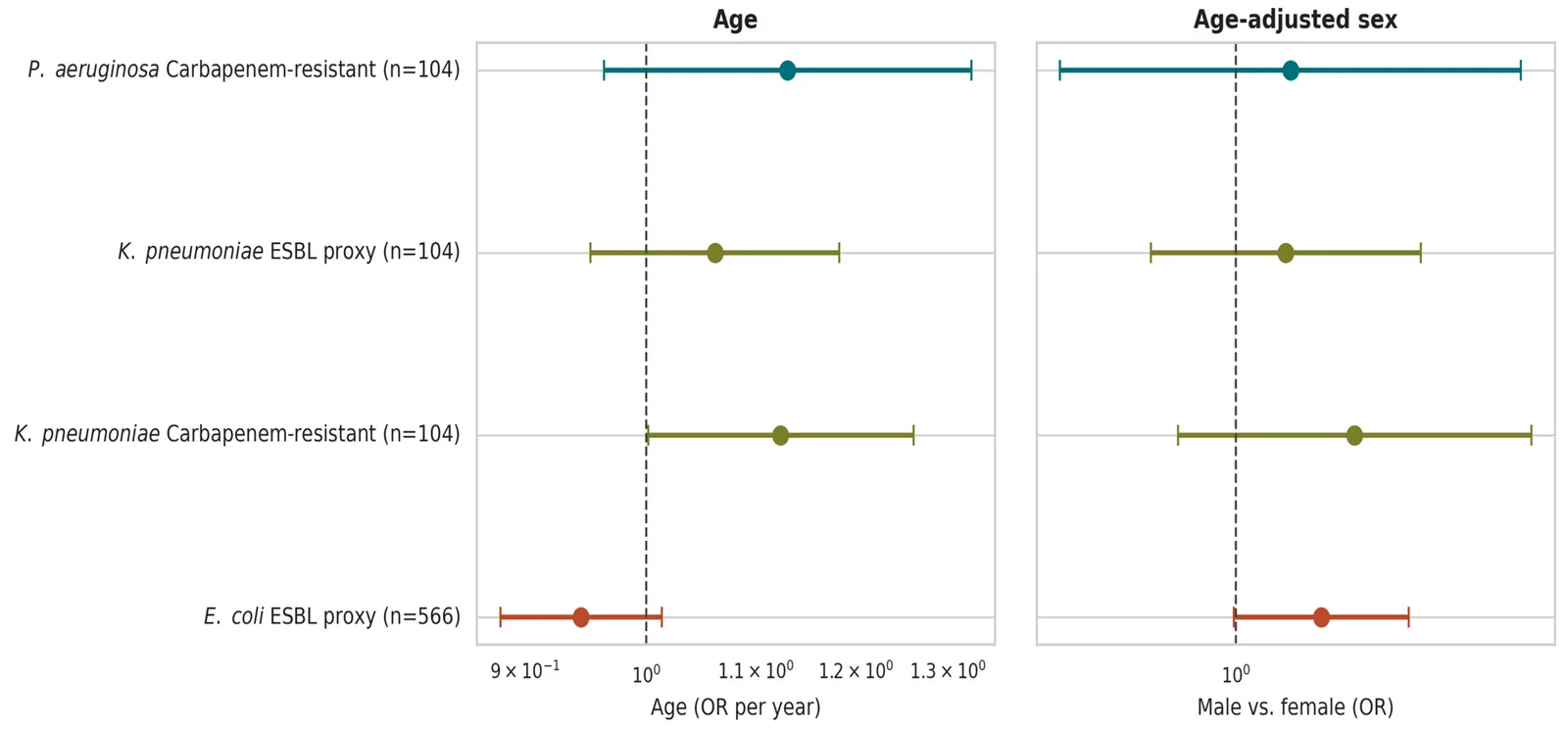
Forest plots of odds ratios for age (per year) and male sex (vs. female) as predictors of presumptive ESBL and carbapenem-resistance phenotypes. Error bars show 95% confidence intervals. Estimates derived from Firth or cluster-robust logistic regression; no associations remained significant after false-discovery-rate correction. Colors identify pathogens: *E. coli* (rust), *K. pneumoniae* (olive), and *P. aeruginosa* (teal); the dashed vertical line denotes OR = 1.

In addition to the descriptive age- and sex-stratified antibiotic results, we modeled age and sex as predictors of the presumptive phenotypes. The models included age as a continuous variable and male sex, with both terms entered together (Figure 3; Supplementary Table S6).

Before FDR correction, male sex showed a weak association with presumptive ESBL in *E. coli* (OR 1.72, 95% CI 0.99–2.99; *p* = 0.055), and age with carbapenem resistance in *K. pneumoniae* (OR 1.12 per year, 95% CI 1.00–1.26; *p* = 0.047). Neither remained significant after correction; q-values were ≥0.177 for age and ≥0.219 for sex across the four phenotype models.

### 2.6. Secondary Temporal Findings

We evaluated temporal resistance among selected antibiotics, testing denominator drift and breakpoint-era sensitivity.

Two trends were significant over the full study period. *E. coli* ampicillin resistance declined from 61.8% in 2015 to 40.6% in 2025 (OR 0.88 per year, 95% CI 0.83–0.94; *p* < 0.0001, q < 0.0001). Trimethoprim-sulfamethoxazole resistance declined from 35.3% to 21.2% (OR 0.88 per year, 95% CI 0.83–0.94; *p* = 0.0001, q = 0.0006). Neither trend showed FDR-significant testing-denominator drift or remained significant in 2021–2025: ampicillin, OR 0.93 per year (95% CI 0.76–1.14; *p* = 0.48), and trimethoprim-sulfamethoxazole, OR 0.93 per year (95% CI 0.74–1.18; *p* = 0.57). These secondary findings should be interpreted cautiously (Supplementary Tables S7–S11). An exploratory full-period model suggested a decline in the *E. coli* presumptive ESBL proxy (OR 0.90 per year, 95% CI 0.84–0.97; *p* = 0.0065, q = 0.0065). Because no post-EUCAST phenotype sensitivity model was prespecified, this was not treated as a main temporal result (Supplementary Table S17).

**Figure 4:**
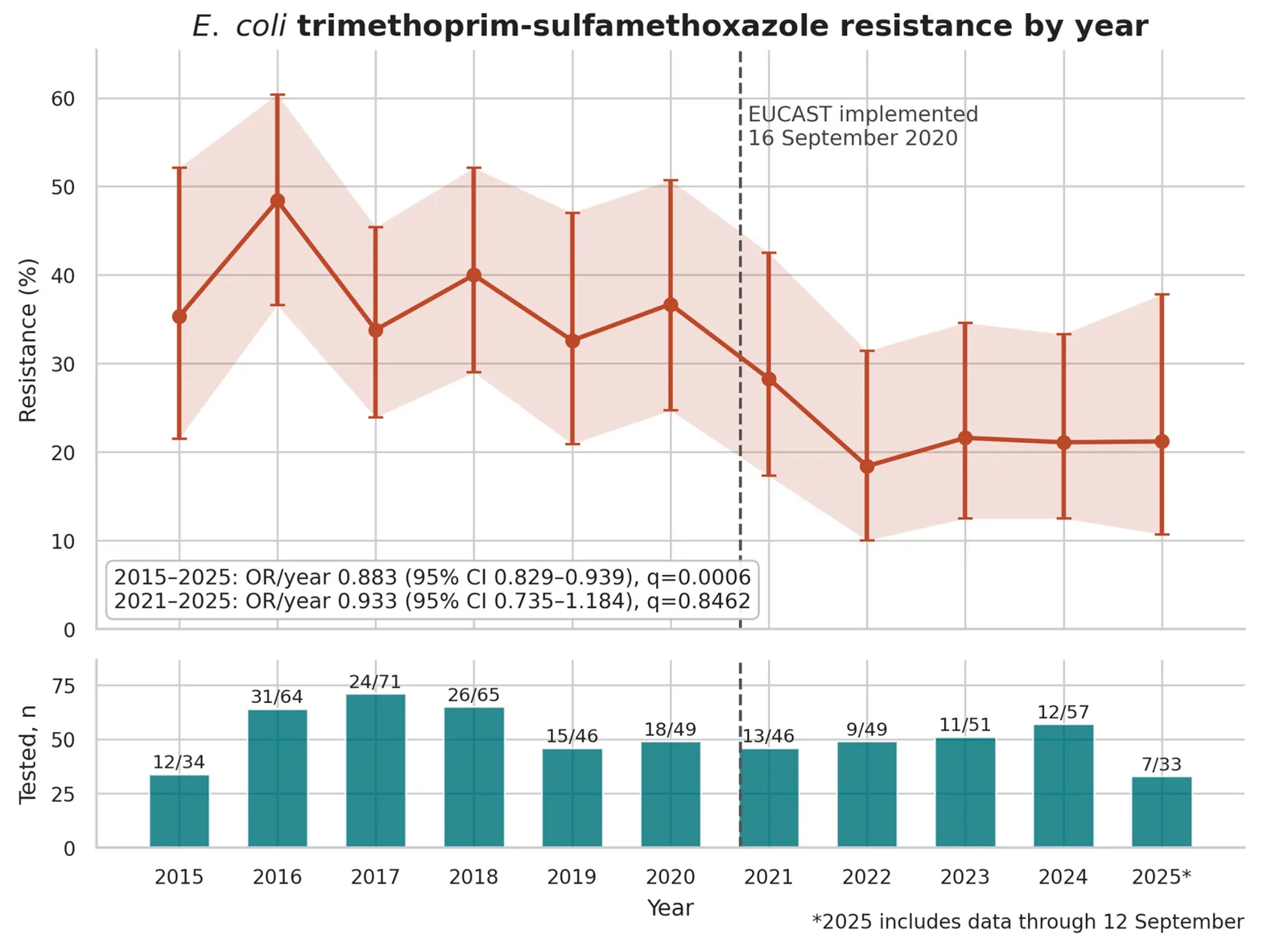
Annual *Escherichia coli* resistance to trimethoprim-sulfamethoxazole. Points and error bars show annual resistance estimates and Wilson 95% confidence intervals; bars show resistant/tested counts. The dashed line marks EUCAST implementation on 16 September 2020. The full-period decline was not confirmed in the complete post-EUCAST period (2021–2025). The shaded band represents the annual Wilson 95% confidence intervals. The asterisk denotes the partial 2025 period, with data through 12 September.

Annual trimethoprim-sulfamethoxazole estimates, tested denominators, and the breakpoint-era transition are shown in Figure 4.

## 3. Discussion

This study describes pediatric urinary resistance epidemiology across three clinically important Gram-negative uropathogens. It shows clear differences in pathogen distribution, phenotype burden and age/sex context. Antimicrobial resistance contributes substantially to global morbidity and mortality, and UTIs are part of that burden [23,24]. Local pediatric urinary susceptibility data therefore remain important for empirical treatment decisions.

Previous Greek pediatric studies reported similar resistance patterns in regional cohorts. Mantadakis et al. found ampicillin susceptibility of 50.5% and nitrofurantoin resistance of 4.4% among pediatric urinary isolates in Thrace [16]. An earlier study from Crete confirmed high ampicillin resistance and preserved nitrofurantoin activity in pediatric urinary isolates [15]. Vazouras et al. reported ampicillin resistance of 42.0% and trimethoprim-sulfamethoxazole resistance of 26.5% in a central Greek district hospital, with third-generation cephalosporins and amikacin retaining high activity [25]. Our findings extend these observations with a broader pathogen spectrum spanning a longer observation window; cephalosporin resistance in *K. pneumoniae* has reached levels that substantially constrain empirical options in this setting.

The increasing relative share of *E. coli* and the declining share of *K. pneumoniae* over the study period may reflect changes in case mix. *K. pneumoniae* is more frequently isolated in infants with congenital anomalies of the kidney and urinary tract, including vesicoureteral reflux [13,26,27], while prior healthcare exposure and antibiotic prophylaxis are established risk factors for ESBL-producing UTI in children [28]. Improved prenatal detection of these anomalies and earlier surgical or prophylactic interventions may have reduced recurrent infections requiring culture in this population, contributing to the declining *K. pneumoniae* share. Even in the absence of such a reduction, a relative increase in community-acquired *E. coli* isolates compared to healthcare-associated non-*E. coli* pathogens would produce the same shift. These pathogen distribution trends are not unique to our setting, but reflect a broader pattern observed across pediatric populations [13].

The most practical message concerns guideline-listed empirical agents. In *E. coli*, ampicillin resistance was high, whereas gentamicin and amikacin resistance remained low. Resistance to cephalosporins was consistently lower than in *K. pneumoniae*, including cefepime, cefixime, cefotaxime, cefpodoxime, ceftazidime, ceftriaxone, and oral and intravenous cefuroxime.

Among non-carbapenem options, nitrofurantoin, amikacin and gentamicin showed the lowest resistance in *E. coli*. Nitrofurantoin remained a highly active oral option for lower UTIs, while gentamicin and amikacin remained active parenteral options. Our finding of near-complete preservation of nitrofurantoin activity aligns with prior literature. The BMJ meta-analysis by Bryce et al. found very low pooled nitrofurantoin resistance in children. Large outpatient U.S. series and previous Greek pediatric data confirm this trend [8,10,16]. By contrast, trimethoprim resistance was near universal and trimethoprim-sulfamethoxazole resistance was also substantial, aligning with prior pediatric outpatient and multidrug-resistance reports [10,11,29]. Their high resistance rates limit their empirical use in this setting.

*K. pneumoniae* showed the highest resistance burden in our dataset. Even its more active selected agents showed higher resistance than the equivalents in *E. coli*. Both presumptive ESBL and carbapenem resistance prevalence were alarmingly high. Published pediatric ESBL UTI cohorts describe a substantial burden of cephalosporin resistance and frequent treatment constraints [17,18,19,20,21,22,28,30,31], while risk factor studies underscore the breadth of concern about multidrug-resistant Enterobacterales in children [32]. Our findings are consistent with the existing literature. They show a heavy local *K. pneumoniae* burden that requires careful management.

*P. aeruginosa* showed comparatively low resistance within the selected antipseudomonal set. Meropenem and piperacillin-tazobactam remained highly active in vitro; however, these estimates arose from a cohort of 105 isolates and used drug-specific tested denominators. Prior pediatric work also supports viewing *P. aeruginosa* UTI as a distinct subgroup within pediatric urinary infection epidemiology [14].

The cohort also showed clear demographic patterning. *E. coli* remained the most common pathogen and had the highest female proportion. *K. pneumoniae* had the youngest age distribution. *P. aeruginosa* had the oldest median age, although its age distribution did not differ significantly from *E. coli*; its sex distribution was more balanced. These patterns add context to the pathogen-specific resistance tables and phenotype burdens. They are also consistent with the broader observation that pediatric urinary microbiology varies with age, sex, setting and case mix [13,16,25].

A pathogen-specific urinary antibiogram is more informative than a general hospital-wide antibiogram when assessing whether narrower agents remain active.

In this cohort, standard empirical options were more consistently active against *E. coli* than against *K. pneumoniae*. The findings support pathogen-specific consultation of the local antibiogram, particularly when *K. pneumoniae* is identified. They do not, however, establish patient-level treatment recommendations because individual care setting, symptoms, comorbidities, prior antimicrobial exposure, and infection severity were unavailable.

Raw age- and sex-stratified resistance percentages varied across several antibiotics. After adjustment for calendar year, age, and sex and correction for multiple testing, increasing age remained associated with *E. coli* trimethoprim-sulfamethoxazole resistance, and male sex remained associated with *E. coli* tobramycin resistance. No age or sex association remained significant in the four presumptive phenotype models. These findings should be interpreted cautiously because clinical history, prior antimicrobial exposure, urinary abnormalities, and care setting were unavailable.

The stewardship implications are practical. Broad-spectrum prescribing for pediatric UTIs has been common for years [9], while prophylactic exposure and recent antibiotic use select for resistant breakthrough infections, including ESBL phenotypes [25,26,27,33,34,35].

Urinary-specific and pediatric syndromic antibiogram approaches have been proposed for this purpose, and decision-support interventions paired with urinary antibiograms can shift prescribing toward narrower-spectrum therapy [36,37,38]. Urine-culture surveillance should be interpreted carefully. It reflects a culture-submitted population. It is not a direct census of all UTI episodes. Culture submission can be selective and biased toward complicated cases [39]. Nevertheless, our data provide a strong local evidentiary basis for stewardship programs.

This study also has methodological strengths. Retrospective microbiology datasets require careful handling. Repeat isolates, selective testing and breakpoint changes can all distort resistance estimates. We therefore validated the patient-level identifier, used a prespecified 90-day episode rule, applied a fixed organism-drug inclusion framework, generated breakpoint-era sensitivity analyses, applied FDR correction, and separated descriptive, phenotype-level and temporal analyses. These measures improve the interpretability of cumulative susceptibility estimates and temporal comparisons. Published studies show that changing from CLSI to EUCAST can materially alter reported susceptibility and multidrug-resistance rates even when the isolate population is unchanged [40,41]. Duplicate-isolate rules can also change cumulative susceptibility estimates through denominator effects [42,43].

This study has several limitations. First, no molecular genotyping was performed. The phenotype definitions are routine AST-based proxy phenotypes, not microbiologically confirmed mechanisms. The presumptive ESBL and carbapenem resistance categories summarize the resistance burden at the phenotype level. They indicate resistance to at least one tested agent within the class, not confirmed resistance across the entire class. Isolates reported as intermediate (CLSI era) or susceptible with increased exposure (EUCAST era) remained in tested denominators but were not counted as resistant. These constructs do not establish molecular ESBL production, carbapenemase production, or specific mechanisms, especially in non-fermenters such as *P. aeruginosa*. Second, in laboratory practice, CLSI criteria were enforced before 16 September 2020 and EUCAST criteria thereafter, so temporal analyses are affected by interpretive drift [40,41]. Third, testing practice changed materially across the study period for several agents, which limits long-term temporal comparisons. Fourth, available demographic variables were limited to age and sex. No data were available on prior antibiotic exposure, urinary anomalies, catheterization, recurrence, prophylaxis, admission status, or healthcare exposure. Unmeasured case-mix differences may therefore contribute to the younger *K. pneumoniae* profile and the observed sex distributions. Fifth, age- and sex-based associations should not be interpreted as strong causal clinical risk factors. Sixth, this cohort represents children who had urine cultures performed, not all pediatric UTI episodes. Culture-based surveillance may overrepresent more complicated or treatment-resistant presentations when culture submission is selective [39]. Individual care setting, admission status, chronic conditions, recurrent UTI, antimicrobial prophylaxis, clinical symptoms, colony counts, mixed-culture assessment, contamination determinations, and indications for culture were unavailable. The isolates could therefore not be classified as symptomatic UTI or asymptomatic bacteriuria.

Finally, temporal analyses reflect changes in reported categorical resistance under evolving standards and testing availability, not harmonized MIC or epidemiological cutoff value trends. Valid retrospective harmonization would require complete MIC or inhibition zone data, test method and AST-card identifiers for every result; these were not consistently retained. The complete post-EUCAST analysis reduced, but could not eliminate, this concern.

## 4. Materials and Methods

### 4.1. Study Design and Dataset

This was a retrospective observational laboratory-based surveillance study of pediatric urinary isolates collected from 2015 through 12 September 2025. The study focused on three predefined Gram-negative urinary pathogens: *E. coli*, *K. pneumoniae*, and *P. aeruginosa*. Only urinary isolates within the study window were included in the final analytic cohort.

Routine microbiology records were exported from the laboratory information system. We included urine cultures submitted under the broad pediatric department code for patients younger than 18 years, reported from 2015 through 12 September 2025, and yielding one of the three study pathogens. Dates, organism labels, and AST fields were standardized before analysis. Patient identifiers were complete for all 776 records, corresponding to 703 individuals (Supplementary Table S12).

The sampling frame comprised children evaluated in the emergency department or admitted to the University Pediatric Department, including the Pediatric Nephrology Service; patients in the pediatric and neonatal intensive care units were not included. All eligible records carried the same broad pediatric department code, so the export did not identify the individual care setting or admission status for each culture. Clinical presentation, underlying chronic conditions, recurrent UTI, antimicrobial prophylaxis, and culture indication were not captured. Eligibility was therefore based on age, study period, and prespecified pathogen. Records with more than one detected organism were retained because mixed-culture and contamination adjudication were unavailable.

The cohort represents laboratory results reported from pediatric urine cultures rather than clinically adjudicated UTI episodes. The study was designed as treatment-oriented categorical susceptibility surveillance, evaluating how the S/I/R interpretations reported at the time of testing constrained or supported treatment options. It was not designed to evaluate longitudinal MIC distributions, epidemiological cutoff value shifts or evolution of resistance mechanisms.

### 4.2. Repeat-Isolate Handling

To accurately represent distinct infectious episodes without overrepresenting repeated cultures, we retained only the first urinary isolate per patient and pathogen in line with standard first-isolate approaches [42], and included subsequent isolates only if they were collected at least 90 days later [43]. This 90-day window was chosen because prior research suggests that episodes separated by this interval likely represent new infecting strains rather than duplicate sampling of the same clinical event [43]. Furthermore, we avoided defining new episodes based solely on changes in susceptibility profiles, as these patterns can fluctuate even during a single infection.

The retained isolate episode was the unit of analysis. Eighteen patients had more than one pathogen-specific urine episode during follow-up. Sensitivity analyses using 30-, 60- and 90-day episode windows each retained all 776 isolates; all-time first-isolate and first-isolate-per-year rules retained 750 and 767 isolates, respectively (Supplementary Table S8).

### 4.3. Antibiotic Selection and Interpretation

Descriptive summaries were limited to EUCAST UTI-relevant organism-drug pairs with at least 20 tested isolates per pathogen. The prespecified antibiotics included oral and intravenous options, and selected antipseudomonal agents.

The analysis retained the categorical S/I/R interpretation reported at the time of testing; missing AST results were not imputed. The laboratory followed annual CLSI updates from M100-S25 to M100-S30 through 15 September 2020, then EUCAST versions 10.0–15.0 (Supplementary Table S16). Historical results were not retrospectively reinterpreted or harmonized. A focused audit confirmed that the *Enterobacterales* trimethoprim-sulfamethoxazole breakpoints did not change across EUCAST versions 10.0–15.0 (MIC: S ≤ 2 and R > 4 mg/L; disk diffusion: S ≥ 14 and R < 11 mm).

Routine AST used the methods and antibiotic panels available at the time of testing. Changes in VITEK 2 (bioMérieux, Marcy-l’Étoile, France) AST-card configurations likely explain much of the antibiotic-specific missingness. Tested denominators are reported by pathogen, antibiotic, and year in Supplementary Table S9, and complete S/I/R distributions in Supplementary Table S13. Reported I results remained in the tested denominator but were not counted as resistant.

Amoxicillin-clavulanate was excluded because its interpretation depends on administration route and clinical indication [44] and our dataset did not distinguish uncomplicated UTI from pyelonephritis or other infections. Cefuroxime was handled by formulation-specific fields: cefuroxime axetil as oral and cefuroxime sodium as intravenous. The reported categories were not retrospectively harmonized.

### 4.4. Presumptive Phenotype Definitions

Phenotypes were defined from routine AST results. Denominators included isolates with at least one valid result for a phenotype-defining antibiotic; isolates were positive if at least one such antibiotic was resistant. Reported I results remained in the denominator but were not counted as resistant. For *E. coli* and *K. pneumoniae*, the presumptive ESBL phenotype was defined by resistance to at least one of cefotaxime, ceftriaxone, ceftazidime, or cefepime, and the carbapenem-resistance phenotype by resistance to at least one of ertapenem, imipenem, or meropenem. For *P. aeruginosa*, carbapenem resistance was defined by imipenem or meropenem resistance. These were presumptive phenotypes, not molecularly confirmed resistance mechanisms.

The presumptive ESBL phenotype was evaluated only for *E. coli* and *K. pneumoniae* because this construct was intended as an *Enterobacterales* cephalosporin resistance proxy. It was not applied to *P. aeruginosa*, for which cephalosporin resistance may reflect distinct intrinsic or acquired mechanisms and is not an appropriate ESBL proxy.

As a stricter sensitivity analysis, phenotype positivity required resistance to at least two tested third- or fourth-generation cephalosporins, with at least two valid results required for inclusion.

### 4.5. Statistical Analysis

Descriptive resistance rates were reported with Wilson 95% confidence intervals. Phenotype prevalence was summarized in the same way. Age was presented descriptively using pediatric age groups [45] and modeled as a continuous variable in supportive phenotype-level logistic models. Sex was assessed as an age-adjusted supportive covariate rather than a headline analytical variable. Temporal logistic regression models were examined separately from the primary descriptive analyses. Firth logistic regression was used as a fallback for sparse or separated data structures. Because many parallel organism-drug comparisons were performed, we controlled the false discovery rate with the Benjamini–Hochberg procedure within prespecified hypothesis families. Temporal signals were interpreted together with denominator trend analyses. Because the laboratory used CLSI criteria before 16 September 2020 and EUCAST criteria thereafter, post-EUCAST sensitivity analyses were generated to assess breakpoint-era robustness.

Age distributions across pathogens were compared using the Kruskal–Wallis test. Pairwise Mann–Whitney tests were adjusted using the Benjamini–Hochberg procedure. Epsilon-squared quantified the omnibus effect size. Sex distribution across pathogens was compared using the chi-square test, with Cramér’s V as the effect size. Both demographic comparisons were repeated after all-time first-isolate filtering as sensitivity analyses.

Antibiotic-specific temporal models used logit(*R*) = *β*_0_ + *β*_1_(*year*). Multivariable antibiotic models used logit(*R*) = *β*_0_ + *β*_1_(*year*) + *β*_2_(*age*) + *β*_3_(*male sex*), and phenotype demographic models used logit(*phenotype*) = *β*_0_ + *β*_1_(*age*) + *β*_2_(*male sex*). Standard logistic regression used patient-clustered robust standard errors when estimable; Firth logistic regression with model-based standard errors was used for sparse or separated data. Complete ORs, 95% CIs, *p*-values, q-values, model methods, and standard-error types are reported in Supplementary Table S6 for phenotype models and Supplementary Table S15 for antibiotic models.

Benjamini–Hochberg correction was applied within pathogen-specific families for antibiotic temporal models (17 *E. coli*, 17 *K. pneumoniae*, and 12 *P. aeruginosa* comparisons), testing-denominator models (25, 15, and 16), unadjusted sex-comparison tests (25, 18, and 15), and multivariable antibiotic models (17, 16, and 11), respectively. Phenotype age and sex coefficients were corrected across the four fitted phenotype models as separate families. Analyses were performed in Python 3.10.13 using pandas 2.2.2, NumPy 1.23.3, SciPy 1.13.1, statsmodels 0.14.2, matplotlib 3.9.1, and seaborn 0.13.2.

## 5. Conclusions

In this pediatric urinary cohort, *E. coli* retained very low resistance to nitrofurantoin and amikacin. *P. aeruginosa* retained low resistance to major antipseudomonal agents. *K. pneumoniae* showed substantially higher cephalosporin resistance. It also demonstrated high presumptive ESBL and carbapenem resistance burdens. The three pathogens differed significantly in age and sex distribution. The youngest age profile was observed in *K. pneumoniae*. The highest female proportion was observed in *E. coli*. These findings detail the local pediatric urinary resistance structure and highlight the challenges of empiric therapy.

## Figures and Tables

**Table 1:** Resistance to selected antibiotics by pathogen. The table includes treatment-relevant agents, phenotype-defining agents, and antibiotics reported in the primary analyses. Columns show isolates tested, isolates resistant, resistance percentage, and Wilson 95% confidence intervals.

Pathogen	Antibiotic	*n* Tested	*n* Resistant	Resistance Rate (%)	95% CI
*E. coli*	Ampicillin	557	298	53.5%	49.3–57.6%
*E. coli*	Cefepime	251	7	2.8%	1.4–5.6%
*E. coli*	Cefixime	25	4	16%	6.4–34.7%
*E. coli*	Cefotaxime	538	68	12.6%	10.1–15.7%
*E. coli*	Cefpodoxime	454	68	15%	12–18.6%
*E. coli*	Ceftazidime	566	53	9.4%	7.2–12%
*E. coli*	Ceftriaxone	39	4	10.3%	4.1–23.6%
*E. coli*	Cefuroxime (IV)	380	60	15.8%	12.5–19.8%
*E. coli*	Cefuroxime (oral)	475	70	14.7%	11.8–18.2%
*E. coli*	Ertapenem	124	2	1.6%	0.4–5.7%
*E. coli*	Imipenem	506	4	0.8%	0.3–2%
*E. coli*	Meropenem	507	2	0.4%	0.1–1.4%
*E. coli*	Amikacin	404	5	1.2%	0.5–2.9%
*E. coli*	Gentamicin	565	44	7.8%	5.9–10.3%
*E. coli*	Tobramycin	500	44	8.8%	6.6–11.6%
*E. coli*	Nitrofurantoin	556	2	0.4%	0.1–1.3%
*E. coli*	Trimethoprim	80	79	98.8%	93.3–99.8%
*E. coli*	Trimethoprim-sulfamethoxazole	565	178	31.5%	27.8–35.4%
*K. pneumoniae*	Cefepime	46	13	28.3%	17.3–42.5%
*K. pneumoniae*	Cefotaxime	100	39	39%	30–48.8%
*K. pneumoniae*	Cefpodoxime	81	34	42%	31.8–52.8%
*K. pneumoniae*	Ceftazidime	104	37	35.6%	27–45.1%
*K. pneumoniae*	Cefuroxime (IV)	78	31	39.7%	29.6–50.8%
*K. pneumoniae*	Cefuroxime (oral)	88	36	40.9%	31.2–51.4%
*K. pneumoniae*	Ertapenem	20	9	45%	25.8–65.8%
*K. pneumoniae*	Imipenem	100	19	19%	12.5–27.8%
*K. pneumoniae*	Meropenem	97	14	14.4%	8.8–22.8%
*K. pneumoniae*	Amikacin	70	6	8.6%	4–17.5%
*K. pneumoniae*	Gentamicin	103	16	15.5%	9.8–23.8%
*K. pneumoniae*	Trimethoprim-sulfamethoxazole	104	40	38.5%	29.7–48.1%
*P. aeruginosa*	Piperacillin-tazobactam	96	2	2.1%	0.6–7.3%
*P. aeruginosa*	Cefepime	76	8	10.5%	5.4–19.4%
*P. aeruginosa*	Ceftazidime	103	9	8.7%	4.7–15.8%
*P. aeruginosa*	Imipenem	90	7	7.8%	3.8–15.2%
*P. aeruginosa*	Meropenem	104	2	1.9%	0.5–6.7%
*P. aeruginosa*	Amikacin	81	6	7.4%	3.4–15.2%
*P. aeruginosa*	Gentamicin	76	7	9.2%	4.5–17.8%
*P. aeruginosa*	Tobramycin	90	7	7.8%	3.8–15.2%

## Data Availability

The underlying microbiology dataset remains subject to institutional and legal data-governance requirements.

## Supplementary Materials

The following supporting information can be downloaded at: https://www.mdpi.com/article/10.3390/antibiotics15080718/s1, Figure S1: Age-group resistance heatmap for Escherichia coli; Figure S2: Age-group resistance heatmap for Klebsiella pneumoniae; Figure S3: Age-group resistance heatmap for Pseudomonas aeruginosa; Figure S4: Annual resistance trends and tested denominators for selected antibiotics in Escherichia coli; Figure S5: Annual resistance trends and tested denominators for selected antibiotics in Klebsiella pneumoniae; Figure S6: Annual resistance trends and tested denominators for selected antibiotics in Pseudomonas aeruginosa; Table S1: Pathogen-mix temporal trends; Table S2: Age- and sex-stratified resistance in Escherichia coli; Table S3: Age- and sex-stratified resistance in Klebsiella pneumoniae; Table S4: Age- and sex-stratified resistance in Pseudomonas aeruginosa; Table S5: Presumptive phenotype burden; Table S6: Phenotype demographic regression models; Table S7: Yearly resistance summary; Table S8: Deduplication sensitivity analysis; Table S9: Testing availability by pathogen, antibiotic, and year; Table S10: Breakpoint-era resistance summary; Table S11: Temporal resistance-trend interpretation; Table S12: Variable dictionary; Table S13: Full S/I/R distribution; Table S14: Strict ESBL-proxy sensitivity analysis; Table S15: Multivariable antibiotic-resistance models; Table S16: Annual CLSI/EUCAST interpretive-standards timeline; Table S17: Exploratory phenotype temporal trends. The supplement provides complete resistance and S/I/R tables; testing availability; stratified, regression, phenotype, temporal, breakpoint-era, and deduplication analyses; the annual CLSI/EUCAST chronology; analysis code; an environment specification; and a variable dictionary.

## Abbreviations

AST	antimicrobial susceptibility testing
CLSI	Clinical and Laboratory Standards Institute
EUCAST	European Committee on Antimicrobial Susceptibility Testing
ESBL	extended-spectrum beta-lactamase
UTI	urinary tract infection
